# Dyadic Mental Health in Paediatric Congenital Heart Disease: Actor–Partner Associations Between Child HRQoL/Depression and Caregiver Stress Across Lesion Severity

**DOI:** 10.3390/healthcare13212681

**Published:** 2025-10-23

**Authors:** Andrada Ioana Dumitru, Adrian Cosmin Ilie, Andrei-Cristian Bondar, Naresh Reddy Mudireddy, Arpan Turimula, Adelina Mavrea, Marioara Boia

**Affiliations:** 1 Doctoral School, Faculty of Medicine, “Victor Babes” University of Medicine and Pharmacy Timisoara, 300041 Timisoara, Romania; andrada.dumitru@umft.ro; 2 Department III Functional Sciences, Division of Public Health and Management, “Victor Babes” University of Medicine and Pharmacy Timisoara, 300041 Timisoara, Romania; ilie.adrian@umft.ro; 3 Faculty of General Medicine, “Titu Maiorescu” University, Calea Văcărești 187, 040051 Bucharest, Romania; 4 Faculty of Medicine, Institute of Medical Sciences, Krishna Vishwa Vidyapeeth “Deemed To Be University”, Karad 415539, India; mudireddynaresh972@gmail.com; 5 Faculty of Medicine, Kaloji Narayana Rao University of Health Sciences, Karad 506007, India; lutherarpan11@gmail.com; 6 Department of Internal Medicine I, Cardiology Clinic, “Victor Babes” University of Medicine and Pharmacy Timisoara, 300041 Timisoara, Romania; mavrea.adelina@umft.ro; 7 Discipline of Neonatology, “Victor Babes” University of Medicine and Pharmacy Timisoara, 300041 Timisoara, Romania

**Keywords:** heart defects, quality of life, child, stress, parents

## Abstract

**Background and Objectives:** We examined how health-related quality of life (HRQoL) in children with congenital heart disease (CHD) and caregiver stress/burnout relate in terms of lesion severity. **Methods:** We enrolled 72 child–caregiver dyads at a tertiary centre (May 2023–April 2025). Children completed PedsQL and CDI (anxiety assessment via SCARED-C was descriptive and not modelled in APIM); caregivers completed SF-36, PSS-10, and the Parental Burnout Assessment (PBA). Lesion severity (mild n = 22, moderate n = 34, severe n = 16) and LVEF were abstracted. Pearson correlations and actor–partner interdependence models (APIM) estimated within- and cross-partner effects. **Results:** Child PedsQL decreased with severity (mild 81.2 ± 7.4; moderate 70.9 ± 8.1; severe 63.3 ± 5.1; *p* < 0.001); caregiver SF-36 Mental Component Summary (MCS) showed a parallel gradient (66.8 ± 9.2; 59.7 ± 8.5; 54.1 ± 7.9; *p* < 0.001). Child HRQoL correlated with caregiver MCS (r = 0.46) and inversely with caregiver stress (PSS r = −0.42) and burnout (PBA r = −0.39). In APIM, caregiver stress predicted a caregiver’s own MCS (actor β = −0.38, *p* < 0.001) and the child’s PedsQL (partner β = −0.26, *p* = 0.002); higher child depressive symptoms predicted lower caregiver MCS (partner β = −0.22, *p* = 0.006). Each step up in lesion severity independently reduced child PedsQL by 7.9 points and caregiver MCS by 5.3 points (both *p* < 0.001). Dyads with unscheduled hospitalizations (n = 43) had poorer scores in both members. **Conclusions:** Psychological wellbeing in CHD dyads is strongly interdependent; caregiver stress relates to lower child HRQoL and child mood to caregiver mental health. Brief dyadic screening (PedsQL/SF-36 with PSS/CDI) and integrated, family-focused interventions may better target high-risk families, particularly with severe lesions or recent hospitalizations.

## 1. Introduction

Congenital cardiac malformations (CCM) represent a persistent global challenge: the 2017 Global Burden of Disease analysis estimated that are ~12 million people living with congenital heart disease (CHD) worldwide and that here has been little change in birth prevalence over three decades, despite a 35% fall in mortality [1]. More recent modelling shows that >217,000 CHD-related deaths still occurred in 2019, with >60% concentrated in low- and middle-income settings [2].

Survival improvements have reframed success away from mortality toward neurodevelopment and life participation. Adults with repaired CHD display elevated rates of attention, memory and executive deficits that erode employability and social integration [3], while paediatric studies now implicate potentially modifiable factors—such as obstructive sleep apnoea—in the neurocognitive lag seen in school-age survivors [4].

Large-cohort work confirms that health-related quality of life (HRQoL) remains 0.5–1 SD below population norms in affected children and adolescents [5,6]. Executive dysfunction and internalising symptoms account for a sizeable share of this variance [7], and multivariable biopsychosocial models explain over half of the between-patient spread, underscoring the importance of psychosocial determinants alongside anatomy [8].

Parents shoulder a parallel—and intertwined—psychological burden. Illness-specific parenting stress, reported in up to half of families referred for behavioural services, rises sharply in single-ventricle physiology [9]. Prospective data show that routine screening flags clinically relevant anxiety or depressive symptoms in one-third of mothers of preschool Fontan patients, leading to calls for systematic HRQoL surveillance within paediatric cardiology pathways [10]. Newer predictive models further demonstrate that parental anxiety and depression independently depress children’s self-reported HRQoL, beyond lesion complexity [11].

Such findings align with dyadic theories of illness adjustment. Actor–partner interdependence studies reveal that a caregiver’s emotion regulation difficulties not only heighten their own distress but also predict their adolescent’s depressive severity [11]; parallel partner effects appear in technology-supported heart-failure dyads [12] and in oncology cohorts where maladaptive coping in one member reverberates in the other’s QoL [13].

Beyond psychosocial sequelae, CCM imposes heavy economic strain. U.S. registry data indicate that families of children with CCM face significantly higher out-of-pocket costs, employment disruption, and unmet mental health needs than other chronic disease groups [14], while recent Romanian work links high perceived stress and parental-burnout scores to lower household income and limited social support [15].

Encouragingly, early, family-centred interventions are emerging. The Dutch CHIP-Family randomised trial demonstrated that a brief psychosocial programme delivered soon after surgery improved parental coping and child behavioural adjustment [16], and a Chinese home-based exercise study reported parallel gains in motor development and parental self-efficacy [17].

Nevertheless, dyadic research in Eastern Europe remains scant. A recent Italian–Romanian protocol underscored the need for longitudinal family-level data but offered no interdependence analyses [18], and qualitative syntheses highlight distinctive coping patterns in post-transition economies [19].

Guided by family systems and dyadic adjustment frameworks, we modelled five attributes central to routine paediatric cardiology follow-up: lesion severity (as an exogenous clinical driver), child health-related quality of life (HRQoL; PedsQL), child depressive symptoms (CDI), caregiver perceived stress (PSS-10), and caregiver mental health-related quality of life (HRQoL; SF-36 MCS). Prior work suggests that greater lesion complexity predicts lower child HRQoL [5,6] and poorer caregiver mental HRQoL through increased clinical complexity and healthcare use [9,10,14]; that parental stress and emotional symptoms depress child-reported HRQoL beyond anatomical factors [10,11]; and that children’s internalising symptoms covary with caregiver distress in dyadic studies, including APIM analyses in chronic disease [13]. Consistent with actor–partner interdependence logic, we therefore hypothesised both cross-partner (“partner”) and within-person (“actor”) effects: higher caregiver stress (PSS-10) would predict lower child HRQoL (PedsQL) [10,11]; higher child depressive symptoms (CDI) would predict lower caregiver mental HRQoL (SF-36 MCS) [13]; caregiver stress would be negatively associated with caregiver MCS [10]; and child depressive symptoms would be negatively associated with child HRQoL [7,8]. We further expected that each step up in lesion severity would independently lower both child HRQoL and caregiver MCS [5,6,9,10], and that recent unplanned hospitalisation would amplify dyadic interdependence by strengthening concordance and cross-partner paths [13].

Accordingly, this study quantified (i) severity-stratified gradients in child HRQoL and caregiver mental health, (ii) within-dyad concordance, and (iii) actor–partner effects between caregiver stress and child HRQoL, and between child depressive symptoms and caregiver mental health. We also explored whether recent unplanned admissions amplify interdependence.

## 2. Materials and Methods

### 2.1. Study Design, Setting and Ethical Oversight

This analytical, cross-sectional investigation conformed to the STROBE recommendations for observational studies and was embedded within routine outpatient visits to the Paediatric Cardiology and Neonatology Departments of “Pius Brînzeu” Emergency Clinical Hospital, Timișoara (a national tertiary referral centre). The tertiary outpatient setting, where families routinely navigate ongoing management rather than perioperative care, aligns with our objective to assess steady-state dyadic interdependence across lesion severity.

Prior to enrolment, the study protocol (IRB #392/25 April 2023) received approval from the institutional ethics committee; all procedures adhered to the 2013 revision of the Declaration of Helsinki, EU GDPR 2016/679, and Romanian Law 190/2018. Written informed consent was obtained from caregivers, and verbal and written assent were obtained from children aged ≥7 years. Participant confidentiality was ensured through anonymized identifier codes and database. Seventy-two child–caregiver dyads met eligibility and completed the protocol.

Null hypotheses: No between-tier differences (ANOVA H0), partner β = 0, actor β = 0, severity β = 0, and no moderation by hospitalisation (Δχ^2^ = 0).

### 2.2. Participants, Recruitment and Sample-Size Justification

Consecutive child–parent dyads attending scheduled follow-up between May 2023 and April 2025 were screened. Inclusion criteria for children were (i) structurally confirmed congenital cardiac malformation (CCM), (ii) age 3–15 years, and (iii) clinical stability (no cardiac surgery <8 weeks). Exclusion criteria comprised genetic syndromes with cognitive disability, insufficient Romanian literacy, and acute intercurrent illness. The accompanying primary caregiver had to have provided ≥6 months of daily care.

**Figure 1:**
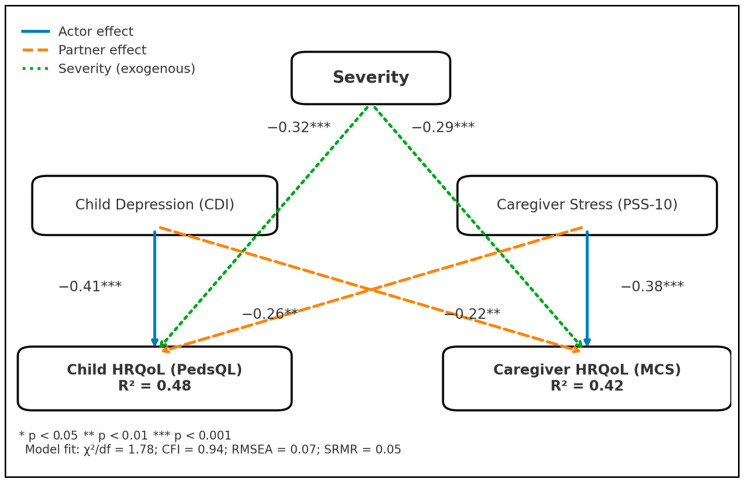
Actor–partner interdependence model (APIM) linking five study attributes. Vertical arrows indicate actor effects (within-person); diagonal arrows indicate partner effects (cross-partner). Lesion severity (AHA complexity tiers) enters as an exogenous predictor of both outcomes. Values are standardised path coefficients from the ML-estimated model (χ^2^/df = 1.78, CFI = 0.94, RMSEA = 0.07, SRMR = 0.05). Outcome nodes display variance explained (R^2^). * *p* < 0.05, ** *p* < 0.01, *** *p* < 0.001.

Congenital disease severity was classified following the American Heart Association complexity scale [20] (simple/mild, moderate, great complexity) and verified independently by two blinded paediatric cardiologists (κ = 0.94). A priori power analysis (G*Power 3.1) indicated that 68 dyads would yield 80% power (α = 0.05) to detect a β = 0.30 partner effect in an actor–partner interdependence model (APIM); seventy-two dyads were finally analysed, allowing ≤10% attrition. While powered for a moderate partner effect (β = 0.30) in APIM, smaller effects may have been missed and confidence intervals should be interpreted accordingly. With a measured-variable APIM (two outcomes, two predictors, covariates), the parameter-to-sample ratio satisfied common heuristics (≥10 cases per free parameter), supporting stable estimation at n = 72. Figure 1 illustrates the resulting APIM specification, with severity modelled as an exogenous predictor of both outcomes.

### 2.3. Clinical Variables

Demographic and medical variables (child age, sex, anatomical diagnosis, most recent left-ventricular ejection fraction [LVEF], and unplanned cardiac admissions during the previous 12 months) were abstracted from electronic health records by investigators blinded to psychosocial data. LVEF represented the arithmetic mean of the two most recent echocardiograms performed ≥3 months apart. Unplanned admission was defined as any emergent hospital stay lasting >24 h for haemodynamic or arrhythmic instability.

### 2.4. Psychosocial Instruments

Operationalization. Child outcomes: HRQoL (PedsQL total) and depressive symptoms (CDI total). Caregiver outcomes: mental health-related QoL (SF-36 MCS), perceived stress (PSS-10), parental burnout (PBA-RO). All questionnaires were available in validated Romanian versions, each displaying strong internal consistency in the present sample (Cronbach α range 0.81–0.93).

The following questionnaires were used in children. PedsQL Scale [21] (child self-report, 23 items): responses on a 5-point Likert scale were reverse-scored and linearly transformed into a 0–100 metric (higher = better HRQoL). Subscale and total scores represent the arithmetic mean of included items; a four-point change is regarded as the minimal clinically important difference (MCID). Children’s Depression Inventory, long form (CDI, 27 items) [22]: each item offers three statements scored 0–2; total range of 0–54. Scores ≥ 19 signify clinically relevant depressive symptomatology. Screen for Child Anxiety-Related Emotional Disorders (SCARED-C, 41 items): scored 0–2, yielding a total 0–82; values ≥ 25 indicate probable anxiety disorder [23].

The following questionnaires were used in adults: Short-Form 36 Health Survey [24] (caregiver, 8 domains): raw domain scores were transformed to norm-based T-scores (mean 50 ± 10) and aggregated into the Mental Component Summary (MCS) and Physical Component Summary (PCS) via factor-weighting algorithms; higher scores denote superior perceived health. Perceived Stress Scale-10 (PSS-10) [25]: items rated 0–4 are summed for a 0–40 total; thresholds are low (0–13), moderate (14–26) and high (27–40) perceived stress. Parental Burnout Assessment [26], Romanian version (PBA-RO, 23 items): items scored 1–5 yield a 23–115 total; scores > 69 mark elevated burnout risk.

Here, “dyadic mental health” denotes measurable, paired psychosocial states in children and their primary caregivers. We indexed child status with HRQoL (PedsQL total) and depressive symptoms (CDI total), and caregiver status with mental health-related quality of life (SF-36 MCS), perceived stress (PSS-10), and parental burnout (PBA-RO). APIM was used to estimate within-person (“actor”) and cross-partner (“partner”) effects.

For ages 5–15 years, children self-reported with staff assistance as needed; for ages 3–4, the validated parent-proxy PedsQL was used, consistent with instrument guidance.

Reporting of measurement content. To enhance transparency, Table 1 details, for each instrument, the construct, domains and number of items, response options, scoring range and interpretation, internal consistency in this sample (Cronbach’s α), and example items. Full item banks are copyrighted by their developers; therefore, we report domain structures and representative items, with complete citations to the original sources [21,22,23,24,25,26]. Romanian validated versions were used for all scales.

### 2.5. Statistical Analysis

Normality was checked with Shapiro–Wilk and Q-Q plots; homogeneity of variance was assessed with Levene’s test. Parametric tests were retained because departures from normality were negligible after inspection of skewness (k = −0.46 to 0.72). Between-group differences in continuous outcomes were examined using one-way ANOVA (severity tiers) or two-way factorial ANOVA with Games–Howell post hoc assessment where variances were unequal. Two-way ANCOVA, adjusted for child age (developmental stage) and LVEF (physiologic reserve), was prespecified based on clinical relevance. Effect sizes are reported as partial η^2^ (<0.01 = small, 0.01–0.06 = medium, >0.14 = large). Bivariate concordance within dyads employed Pearson’s r. Dyadic influence was modelled through APIM with maximum-likelihood estimation; model fit was deemed acceptable if χ^2^/df < 3, CFI > 0.90 and RMSEA < 0.08, and SRMR ≤ 0.08. Moderation by hospitalisation was explored with multi-group APIM and the comparison of nested models via Δχ^2^ tests. Two-tailed *p*-values < 0.05 defined statistical significance; the Benjamini–Hochberg false-discovery rate controlled for multiplicity (q = 0.10). Analyses were run in R (v4.3.x, R Foundation); ANOVA/ANCOVA and correlations used base stats and car; effect sizes were fit with effectsize; and APIM was fit via lavaan (maximum likelihood). Figures were generated with ggplot2.

## 3. Results

Table 2 quantifies the structural features of the cohort with precise numeric granularity. Children’s mean age was 7.9 ± 3.0 years (range 3–15); 38 of 72 participants were boys, yielding a male proportion of 52.8%. Caregivers averaged 36.5 ± 7.2 years (range 23–54) and were predominantly mothers—53 mothers versus 19 fathers. This corresponded to 73.6% maternal representation. Lesion stratification followed the AHA complexity grid: 22 children (30.6%) had mild malformations, 34 (47.2%) moderate malformations and 16 (22.2%) severe malformations. Left-ventricular ejection fraction remained largely preserved across the sample, with a mean of 61.7 ± 7.7% and an interquartile span from 57% to 67%. Clinical instability was frequent: 43 dyads (59.7%) recorded at least one unplanned cardiac admission exceeding 24 h in the preceding 12 months, while the remaining 29 (40.3%) avoided emergent hospitalisation.

Table 3 reports four continuous psychosocial outcomes across mild (n = 22), moderate (n = 34) and severe (n = 16) lesion groups, accompanied by omnibus one-way ANOVA *p*-values. Child PedsQL totals declined from 81.2 ± 7.4 in the mild tier to 70.9 ± 8.1 in the moderate tier and reached 63.3 ± 5.1 in the severe tier (F = 56.8, *p* < 0.001). Caregiver SF-36 Mental Component Summary mirrored this gradient: 66.8 ± 9.2, 59.7 ± 8.5 and 54.1 ± 7.9, respectively (F = 18.5, *p* < 0.001). Children’s depressive scores rose sharply—CDI 9.4 ± 3.1, 13.7 ± 3.9, and 18.1 ± 2.6 (F = 63.4, *p* < 0.001)—while caregiver stress escalated in parallel: PSS-10 18.7 ± 5.1, 22.9 ± 5.6, and 26.3 ± 6.0 (F = 17.9, *p* < 0.001). Post hoc Games–Howell tests showed every pairwise difference significant at q < 0.10 for all four metrics, with mean PedsQL decrements of 10.3 points between mild and moderate and 7.6 points between moderate and severe, both surpassing the four-point minimal clinically important threshold.

Table 4 lists four Pearson correlation coefficients with associated two-tailed *p*-values (N = 72). Child PedsQL correlated positively with caregiver SF-36 MCS at r = 0.46 (*p* < 0.001), indicating that 21% of variance in one metric covaried with the other (r^2^ = 0.21). An inverse correlation was observed between child PedsQL and caregiver PSS-10 (r = −0.42, *p* = 0.001), and between caregiver PBA total and child PedsQL (r = −0.39, *p* = 0.001), explaining 17% and 15% of shared variance, respectively. Caregiver SF-36 MCS and child CDI displayed r = −0.38 (*p* = 0.002), translating to r^2^ = 0.14. All correlations met the Benjamini–Hochberg-adjusted significance threshold (q = 0.10). No coefficient exceeded the ±0.50 boundary, situating the dyadic concordance firmly in the moderate effect-size band while underscoring statistically robust bidirectional linkages across quality-of-life, stress and mood domains.

Table 5 summarises five standardised path estimates derived from maximum-likelihood APIM (χ^2^/df = 1.78, CFI = 0.94, RMSEA = 0.07). Actor effects were largest: child CDI on child PedsQL β = −0.41 (SE = 0.08, z = −5.09, *p* < 0.001) and caregiver PSS-10 on caregiver MCS β = −0.38 (SE = 0.09, z = −4.36, *p* < 0.001). Partner pathways retained significance: caregiver PSS-10 predicting child PedsQL β = −0.26 (SE = 0.08, z = −3.16, *p* = 0.002) and child CDI predicting caregiver MCS β = −0.22 (SE = 0.08, z = −2.75, *p* = 0.006). Lesion severity exerted independent negative effects on both outcomes, β = −0.32 (child) and β = −0.29 (caregiver), each *p* < 0.001. Together, the model explained 48% of variance in child PedsQL and 42% in caregiver MCS, indicating substantial dyadic coupling after controlling for anatomical complexity.

Consistent with H1c–H1d, actor paths were largest (child CDI → child PedsQL; caregiver PSS → caregiver MCS). In line with H1a–H1b, both partner paths were significant and negative (caregiver stress lowering child HRQoL; child depression lowering caregiver MCS). H2 was supported by independent, stepwise severity effects on both outcomes.

Table 6 presents mean z-standardised discrepancies (child PedsQL minus caregiver MCS) with 95% confidence intervals and one-sample *t*-test *p*-values for deviation from zero. Mild lesions showed a discrepancy of −0.12 (95% CI −0.41 to 0.17; t = −0.83, df = 21, *p* = 0.417). In moderate lesions, the mean gap widened to −0.38 (CI −0.63 to −0.13; t = −3.14, df = 33, *p* = 0.004). Severe lesions displayed the greatest divergence at −0.66 (CI −1.02 to −0.30; t = −4.13, df = 15, *p* < 0.001). Standard deviations were 0.54, 0.61 and 0.58, respectively, evidencing comparable intra-group dispersion. Linear trend analysis across tiers produced β = −0.27 per severity step (SE = 0.06, *p* < 0.001), confirming a monotonic increase in negative dyadic discrepancy as complexity escalates.

Table 7 compares psychosocial means between dyads without (n = 29) and with (n = 43) unplanned admissions. Children’s PedsQL averaged 76.5 ± 10.4 versus 69.1 ± 8.2 (t = 3.13, df = 70, *p* = 0.003, Cohen d = 0.78). Caregiver MCS was 63.4 ± 8.6 versus 57.2 ± 8.1 (t = 3.20, df = 70, *p* = 0.002, d = 0.75). The dyadic PedsQL-MCS correlation coefficient rose from r = 0.32 (95% CI 0.00–0.57) in the no-admission group to r = 0.55 (CI 0.30–0.73) post-admission; Fisher’s z-test yielded z = 1.39, *p* = 0.083, indicating a non-significant yet appreciable increase in interdependence magnitude.

Table 8 details mean ± SD values for PedsQL and CDI across six sex-severity cells, plus two-way ANOVA results. Boys’ PedsQL scores were 83.1 ± 6.8 (mild), 73.2 ± 7.9 (moderate) and 65.2 ± 4.9 (severe); corresponding figures for girls were 79.0 ± 7.6, 68.1 ± 7.8 and 61.7 ± 5.0. Boys’ CDI means were 8.6 ± 2.9, 12.7 ± 3.6 and 17.2 ± 2.4; girls’ means were 10.3 ± 3.2, 14.9 ± 3.8 and 18.9 ± 2.5. Main-effect F-statistics were as follows: severity F(2,66) = 55.2 for PedsQL (η^2^ = 0.45) and 68.0 for CDI (η^2^ = 0.51); sex F(1,66) = 6.8 for PedsQL (η^2^ = 0.09) and 8.1 for CDI (η^2^ = 0.11). Interaction terms were non-significant (PedsQL F = 1.5, *p* = 0.23; CDI F = 2.0, *p* = 0.14). Welch *t*-tests confirmed within-tier sex gaps >4 PedsQL points and >2 CDI points in all but mild CDI (*p* = 0.06).

**Figure 2:**
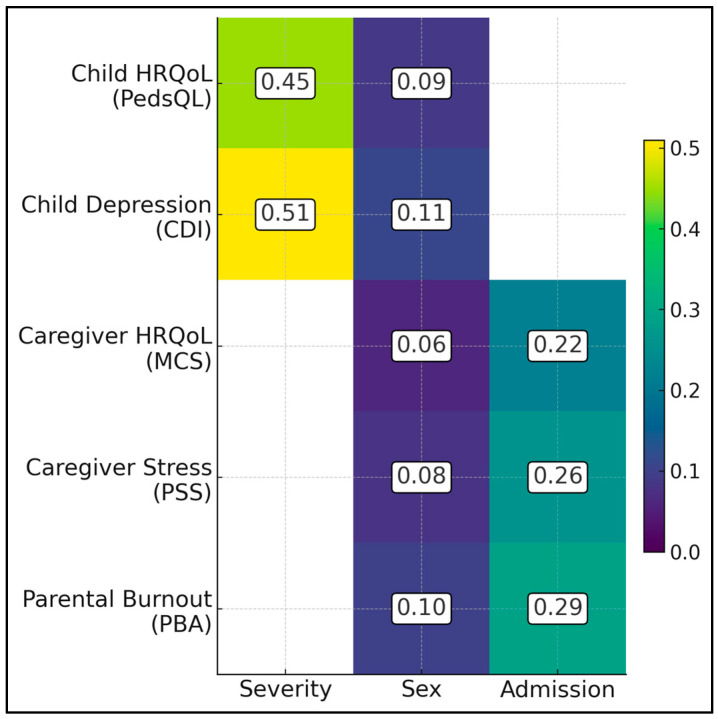
Heat-map of effect sizes.

Table 9 comprises four sex-by-admission subgroups: mothers without admissions (n = 19), mothers with admissions (n = 34), fathers without (n = 10) and fathers with (n = 9). SF-36 MCS means were 62.5 ± 8.4, 56.2 ± 7.8, 66.1 ± 8.8 and 60.4 ± 7.5, respectively. PSS-10 means were 19.3 ± 5.0, 24.2 ± 5.4, 16.8 ± 4.8 and 21.1 ± 5.1. PBA-RO totals were 55.8 ± 11.2, 66.4 ± 12.8, 52.1 ± 10.4 and 60.3 ± 11.5. Two-way ANOVA yielded strong main effects of admission: MCS F(1,68) = 18.2 (η^2^ = 0.22), PSS F = 22.7 (η^2^ = 0.26), and PBA F = 26.5 (η^2^ = 0.29). Sex effects were smaller yet significant: MCS F = 4.3 (η^2^ = 0.06), PSS F = 5.6 (η^2^ = 0.08), and PBA F = 6.9 (η^2^ = 0.10). Interaction terms remained non-significant (all F < 1.0, *p* > 0.40), indicating additive rather than multiplicative burdens (Figure 2).

## 4. Discussion

### 4.1. Literature Findings

Our dyadic approach extends prior single-centre Romanian research by demonstrating that parental stress not only parallels but predicts their child’s quality of life, even after adjusting for anatomical severity. Partner effects of comparable magnitude have been reported in cystic fibrosis and oncology but seldom quantified in paediatric cardiology, underscoring the novel contribution of this study.

Reciprocity was bidirectional: child depression exerted a measurable impact on caregiver mental health, supporting systemic family theories that view illness as a shared experience. The stronger concordance among hospitalised dyads suggests acute episodes heighten emotional synchrony, potentially rendering families more receptive to joint psychosocial support during transition-of-care clinics.

Practically, findings argue for integrated screening—PedsQL and SF-36 for functional outlook, PSS-10 and CDI for mood—at every annual review. Interventions such as dyadic cognitive–behavioural therapy, mindfulness-based stress reduction for parents alongside age-tailored coping skills for children, or structured family exercise programmes could disrupt the negative feedback loop identified in APIM. Future trials should test whether targeting either partner preferentially yields ripple benefits across the dyad.

Our findings extend the international literature by reaffirming the steep HRQoL gradient across anatomical complexity while adding a dyadic lens. The 17-point PedsQL gap we observed between mild and severe lesions is almost identical to the 15-point decrement reported in a recent Brazilian case–control study that compared 70 children with diverse CHD to healthy peers and showed that lower functional fitness and reduced engagement in daily activities co-segregated with poorer HRQoL—reinforcing the idea that physiological limitation and psychosocial wellbeing travel together regardless of setting or methodology [20].

Sex-stratified analyses revealed that girls scored ~4–5 points lower on PedsQL and ~2 points higher on depressive symptoms than boys within each severity tier, with medium effect sizes even after age adjustment. This mirrors data from a multicentre Jordanian–Qatari cohort of 552 Arab children, where female sex independently predicted poorer child-reported total and psychosocial PedsQL despite similar parental ratings. Taken together, these studies suggest a small but consistent vulnerability in girls that may reflect gendered coping styles or differential parental expectations rather than lesion biology, warranting sex-responsive psychological screening protocols [27].

The amplification of dyadic distress after unplanned admission echoes work in neonatal and early-infancy CHD contexts. Australian survey data collected three to seven months post-surgery showed that longer intensive-care exposure was associated with higher maternal stress and weaker mother–infant bonding, which in turn predicted suboptimal parenting self-efficacy [28,29]. Physiological biomarkers strengthen this link: a U.S. cohort demonstrated that flatter salivary–cortisol slopes shortly after discharge forecasted persistent post-traumatic stress symptoms at three months, underscoring how acute clinical events can leave a measurable biological footprint that perpetuates family-level psychosocial risk.

Our actor–partner paths align with dyadic evidence outside cardiology. In a pan-diagnostic Australian sample, unmet parental supportive-care needs and child emotional dysfunction jointly accounted for one-third of the variance in parental depression and anxiety, confirming that caregivers’ wellbeing hinges on their child’s internalising burden. Moreover, a Chinese actor–partner interdependence mediation model in 341 adolescent–parent dyads with chronic illness showed that family resilience exerted both direct benefits and indirect effects through dyadic coping on each partner’s adjustment, paralleling our observation that caregiver stress reverberates in child HRQoL and vice versa [30,31].

Parental burnout emerged as an independent correlate of child HRQoL, supporting broader population data in which burnout surpassed depression, anxiety, and even “dark-triad” traits as the strongest behavioural risk factor for neglect and harsh parenting. Encouragingly, a 2024 JAMA Pediatrics meta-analysis covering 15 RCTs showed that structured problem-solving-skills training (PSST) delivered to parents of children with chronic conditions produced moderate reductions in caregiver stress (Hedges g ≈ 0.45) and small but significant improvements in child psychosocial functioning, suggesting a readily scalable intervention to break the feedback loop we identified [32,33,34].

Finally, digital innovation offers new avenues for reaching high-burden families in resource-limited settings such as ours. The HEARTPrep programme, co-designed with parents and delivered via a mobile app plus brief telehealth coaching, demonstrated high usability and acceptability among mothers carrying a foetus with CHD and is now moving toward efficacy testing. Embedding similar hybrid platforms into Romanian follow-up pathways could provide just-in-time stress-management tools, enable remote monitoring of PSS/CDI scores and facilitate triage to in-person services, thereby operationalising the family-centred model our data advocate.

Family roles, stigma around mental health, and access pathways in Romania and neighbouring post-transition settings may amplify caregiver burden and dampen help-seeking. These cultural moderators warrant explicit measurement (perceived stigma, social support) in future dyadic models. Moreover, external pressures—financial strain, employment instability, transport burden, and school absenteeism—likely intensify dyadic coupling after acute events. Including these stressors as moderators may sharpen risk stratification. Intra-family processes (communication quality, co-rumination, emotion regulation) plausibly mediate partner effects; validated brief measures could be embedded without overloading visits.

Routine screening should include a brief financial hardship item to trigger social-work referral, given the known link between economic stress and parental burnout. The cross-sectional interdependence observed here motivates multi-wave cohorts to map trajectories before and after clinical inflection points (such as surgery, decompensation). Quarterly, alternating assessments (PedsQL/SF-36, PSS-10/CDI) over 12–18 months could test whether spikes in one partner forecast subsequent decrements in the other (cross-lagged APIM), and whether these effects are strongest near acute events. Embedding brief family-focused interventions at transition clinics would allow randomised, mechanistic tests of whether reducing caregiver stress propagates gains in child HRQoL (and vice versa).

Findings support reciprocal-influence accounts in paediatric chronic illness, indicating that caregiver stress is not merely a correlate but a plausible mechanistic driver of child HRQoL deficits, and vice versa. This strengthens the case for family systems-informed care pathways in CHD.

### 4.2. Study Limitations

The single-centre, n = 72 design constrains external validity and precision, particularly for subgroup contrasts; however, lesion distribution mirrors national registry patterns. Cross-sectional data preclude causal inference, though APIM strengthens directional plausibility. Self-report bias remains possible, especially with psychologist assistance for younger children. We lacked socioeconomic and marital-quality variables that may moderate dyadic dynamics. Additional unmeasured factors could bias estimates, including time since diagnosis, socioeconomic resources, marital relationship quality, parent and child comorbidities, and cultural coping norms. Moreover, child self-report comprehensibility varies at younger ages; although instrument guidance was followed (proxy for ages 3–4; assisted self-report ≥ 5 years), residual measurement error is plausible. Parental and child multimorbidity (e.g., parental depression, child neurodevelopmental conditions) was not ascertained and could confound or mediate observed links. Finally, structural-equation modelling assumes linearity; complex, non-linear interactions might be underestimated.

## 5. Conclusions

In paediatric CHD, child HRQoL and caregiver mental health move together. Beyond anatomical severity, higher caregiver stress relates to lower child HRQoL, while greater child depressive symptoms relate to poorer caregiver mental health. Brief, routine dyadic screening (PedsQL/SF-36 with PSS-10/CDI) can flag families at risk—especially after recent hospitalizations—and guide family-focused supports within cardiology follow-up.

## Figures and Tables

**Table 1:** Measurement overview, response formats, scoring and example items.

Instrument (Construct)	Domains (Items)	Response Options	Score Range & Direction	Example Item(s) *	Internal Consistency (This Sample)
PedsQL 4.0 (Child HRQoL) [21]	Physical (8), Emotional (5), Social (5), School (5); Total (23)	0–4 (“never” to “almost always”) → reverse-scored and linearly transformed to 0–100	Higher = better HRQoL; MCID ≈ 4 points	“It is hard for me to run.”; “I feel sad or blue.”	α_total = 0.91 (subscales 0.85–0.90)
CDI (27-item) (Child depression) [22]	Dysphoria, Negative self-esteem, etc. (total score)	0–2 (three statements per item)	0–54; ≥19 suggests clinically relevant symptoms	“I am sad once in a while/many days/all the time.”	α = 0.88
SCARED-C (screen; descriptive) [23]	Panic/somatic, GAD, Separation, Social, School (41)	0–2	0–82; ≥25 indicates probable anxiety disorder	“I worry about things working out for me.”	α = 0.93
SF-36 (caregiver)—MCS used [24]	8 domains aggregated to MCS	Item-specific Likert formats; norm-based scoring	T-scores (mean 50, SD 10); higher = better	“Have you felt downhearted and blue?”	α_MCS = 0.86
PSS-10 (caregiver stress) [25]	Perceived stress (10)	0–4 (“never” to “very often”)	0–40; 0–13 low, 14–26 moderate, 27–40 high	“In the last month, how often have you felt nervous and ‘stressed’?”	α = 0.84
PBA-RO (parental burnout) [26]	Exhaustion, Contrast with previous self, Saturation, Emotional distancing (23)	1–5	23–115; >69 elevated risk	“I feel exhausted by my role as a parent.”	α = 0.87

* Illustrative examples only; full proprietary wording is available in the cited manuals.

**Table 2:** Demographic and clinical characteristics of 72 child–parent dyads.

Variable	Value
Child age, years	7.9 ± 3.0
Male children	38 (52.8%)
Caregiver age, years	36.5 ± 7.2
Mothers/Fathers	53/19
Severity tier	Mild 22 (30.6%)
	Moderate 34 (47.2%)
	Severe 16 (22.2%)
Mean LVEF, %	61.7 ± 7.7
≥1 unplanned admission (12 months)	43 (59.7%)

**Table 3:** Mean psychosocial scores by severity tier.

Metric	Mild (n = 22)	Moderate (n = 34)	Severe (n = 16)	*p*
Child PedsQL, total	81.2 ± 7.4	70.9 ± 8.1	63.3 ± 5.1	<0.001
Caregiver SF-36 MCS	66.8 ± 9.2	59.7 ± 8.5	54.1 ± 7.9	<0.001
Child CDI	9.4 ± 3.1	13.7 ± 3.9	18.1 ± 2.6	<0.001
Caregiver PSS-10	18.7 ± 5.1	22.9 ± 5.6	26.3 ± 6.0	<0.001

Values are mean ± SD. One-way ANOVA across lesion tiers with Games–Howell post hoc when variances were unequal; Benjamini–Hochberg FDR set at q = 0.10 for pairwise contrasts. Reported *p* is omnibus ANOVA (two-tailed).

**Table 4:** Child–parent concordance.

Pairing	*r*	*p*
Child PedsQL-Caregiver SF-36 MCS	0.46	<0.001
Child PedsQL-Caregiver PSS-10	−0.42	0.001
Caregiver MCS-Child CDI	−0.38	0.002
Caregiver PBA total-Child PedsQL	−0.39	0.001

**Table 5:** Actor–partner interdependence model (standardised β, n = 72).

Predictor	Outcome (Child PedsQL)	Outcome (Caregiver MCS)
Child CDI (actor)	−0.41 ***	–
Caregiver PSS (partner)	−0.26 **	–
Caregiver PSS (actor)	–	−0.38 ***
Child CDI (partner)	–	−0.22 **
Severity tier	−0.32 ***	−0.29 ***

*** *p* < 0.001; ** *p* < 0.01 model fit: χ^2^/df = 1.78; CFI = 0.94; RMSEA = 0.07; SRMR = 0.05.

**Table 6:** Severity-stratified dyadic discrepancy (child PedsQL–caregiver MCS, z-standardised).

Tier	Mean Discrepancy	95% CI	*p* vs. 0
Mild	−0.12	−0.41; 0.17	0.417
Moderate	−0.38	−0.63; −0.13	0.004
Severe	−0.66	−1.02; −0.30	<0.001

**Table 7:** Impact of recent hospitalisation on dyadic scores.

Metric	0 Admissions (n = 29)	≥1 Admission (n = 43)	*p*
Child PedsQL	76.5 ± 10.4	69.1 ± 8.2	0.003
Caregiver MCS	63.4 ± 8.6	57.2 ± 8.1	0.002
Dyadic correlation (PedsQL-MCS)	0.32	0.55	–

**Table 8:** Child psychosocial outcomes by sex and lesion severity (mean ± SD).

Outcome	Mild	Moderate	Severe	Two-Way ANOVA (Main Effects & Interaction)
PedsQL total	Boys 83.1 ± 6.8	Boys 73.2 ± 7.9	Boys 65.2 ± 4.9	Sex F = 6.8, *p* = 0.011, η^2^ = 0.09
	Girls 79.0 ± 7.6	Girls 68.1 ± 7.8	Girls 61.7 ± 5.0	Severity F = 55.2, *p* < 0.001, η^2^ = 0.45
				Sex × Severity F = 1.5, *p* = 0.23
CDI total	Boys 8.6 ± 2.9	Boys 12.7 ± 3.6	Boys 17.2 ± 2.4	Sex F = 8.1, *p* = 0.006, η^2^ = 0.11
	Girls 10.3 ± 3.2	Girls 14.9 ± 3.8	Girls 18.9 ± 2.5	Severity F = 68.0, *p* < 0.001, η^2^ = 0.51
				Sex × Severity F = 2.0, *p* = 0.14

Within-tier sex differences tested with Welch *t*-tests (all *p* < 0.05 except mild CDI, *p* = 0.06).

**Table 9:** Caregiver mental-health metrics by caregiver sex and child hospitalisation status (mean ± SD).

Metric	Mothers, 0 Admissions (n = 19)	Mothers, ≥1 Admission (n = 34)	Fathers, 0 Admissions (n = 10)	Fathers, ≥1 Admission (n = 9)	Two-Way ANOVA (Main Effects & Interaction)
SF-36 MCS	62.5 ± 8.4	56.2 ± 7.8	66.1 ± 8.8	60.4 ± 7.5	Admission F = 18.2, *p* < 0.001, η^2^ = 0.22
					Sex F = 4.3, *p* = 0.042, η^2^ = 0.06
					Sex × Admission F = 0.6, *p* = 0.44
PSS-10	19.3 ± 5.0	24.2 ± 5.4	16.8 ± 4.8	21.1 ± 5.1	Admission F = 22.7, *p* < 0.001, η^2^ = 0.26
					Sex F = 5.6, *p* = 0.020, η^2^ = 0.08
					Sex × Admission F = 0.7, *p* = 0.40
PBA-RO total	55.8 ± 11.2	66.4 ± 12.8	52.1 ± 10.4	60.3 ± 11.5	Admission F = 26.5, *p* < 0.001, η^2^ = 0.29
					Sex F = 6.9, *p* = 0.010, η^2^ = 0.10
					Sex × Admission F = 0.5, *p* = 0.48

## Data Availability

The dataset used and analyzed in this study is available from the corresponding author. The data are not publicly available due to privacy concerns and ethical restrictions.
